# Identification of Potential Biomarkers and Immune Infiltration Characteristics in Ulcerative Colitis by Combining Results from Two Machine Learning Algorithms

**DOI:** 10.1155/2022/5412627

**Published:** 2022-08-01

**Authors:** Minchun Bu, Xiandong Cao, Bo Zhou

**Affiliations:** Department of General Surgery, First Affiliated Hospital of Anhui Medical University, Hefei, 230022 Anhui, China

## Abstract

**Objective:**

This study was designed to identify potential biomarkers for ulcerative colitis (UC) and analyze the immune infiltration characteristics in UC.

**Methods:**

Datasets containing human UC and normal control tissues (GSE87466, GSE107597, and GSE13367) were downloaded from the GEO database. Then, the GSE87466 and GSE107597 datasets were merged, and the differentially expressed genes (DEGs) between UC and normal control tissues were screened out by the “limma R” package. The LASSO regression model and support vector machine recursive feature elimination (SVM-RFE) were performed to screen out the best biomarkers. The GSE13367 dataset was used as a validation cohort, and the receiver operating characteristic curve (ROC) was used to evaluate the diagnostic performance. Finally, the immune infiltration characteristics in UC were explored by CIBERSORT, and we further analyzed the correlation between potential biomarkers and different immune cells.

**Results:**

A total of 76 DEGs were screened out, among which 56 genes were upregulated and 20 genes were downregulated. Functional enrichment analysis revealed that these DEGs were mainly involved in immune response, chemokine signaling, IL−17 signaling, cytokine receptor interactions, inflammatory bowel disease, etc. ABCG2, HSPB3, SLC6A14, and VNN1 were identified as potential biomarkers for UC and validated in the GSE13367 dataset (AUC = 0.889, 95% CI: 0.797~0.961). Immune infiltration analysis by CIBERSORT revealed that there were significant differences in immune infiltration characteristics between UC and normal control tissues. A high level of memory B cells, *γδ* T cells, activated mast cells, M1 macrophages, neutrophils, etc. were found in the UC group, while a high level of M2 type macrophages, resting mast cells, eosinophils, CD8+ T cells, etc. were found in the normal control group.

**Conclusion:**

ABCG2, HSPB3, SLC6A14, and VNN 1 were identified as potential biomarkers for UC. There was an obvious difference in immune infiltration between UC and normal control tissues, which may provide help to guide individualized treatment and develop new research directions.

## 1. Introduction

Ulcerative colitis (UC) is a chronic inflammatory bowel disease that can affect any part of colorectum [1–3]. It starts with inflammation of the rectal mucosa and expands proximally in a continuous manner with varying degrees of disease [2]. Globally, the incidence of UC is on the rise, and its proportion in developing countries has increased [4]. UC is characterized by alternating relapse and remission processes, and the typical symptoms of patients are mucinous pus and blood in the stool, abdominal pain, urgency of stool and tenesmus [2, 3]. Due to the inflammatory nature of UC, if improperly treated, it can cause continuous intestinal damage and even further increase the risk of colorectal cancer [3, 4]. UC is also a complex multifactorial disease, and the specific pathogenesis of UC remains unclear, it may be caused by the chronic intestinal mucosal injury induced by the interaction between genetic and environmental factors and then the imbalance of immune system [5]. Some studies [4, 5] have shown that UC is characterized by adaptive immune system imbalance, especially the imbalance between regulatory T cells and T-helper (Th) 2 cells: Th2 reaction can activate the natural killer T cells in colorectum, which then secrete a large number of cytokines, inducing epithelial cell apoptosis and blocking tight junction; meanwhile, these cytokines can stimulate the expression of adhesion molecules of vascular endothelial cell, thereby promoting leukocyte adhesion and extravasation into tissues, resulting in intestinal inflammation and injury. At present, the treatment is mainly through diet control and symptomatic treatment of drugs such as aminosalicylic acid and glucocorticoids, which has some limitations; meanwhile, there were also no specific markers for UC to assess the prognosis [2, 3, 6].

In recent years, gene expression microarray research has been widely used in the exploration of potential biomarkers for complex diseases, so as to further analyze its pathogenesis and find new therapeutic targets [7, 8]. In our study, we selected 3 datasets (GSE87466, GSE107597, and GSE13367) in the Gene Expression Comprehensive (GEO) database. Merged the GSE87466 and GSE107597 datasets, two machine learning algorithms were performed to screen out the best feature genes that can distinguish UC from the normal tissues. Finally, feature genes were identified and validated in the GSE13367 dataset, and CIBERSORT [9] was used to analyze the immune infiltration characteristics in UC.

## 2. Materials and Methods

### 2.1. Data Preparation

**Figure 1:**
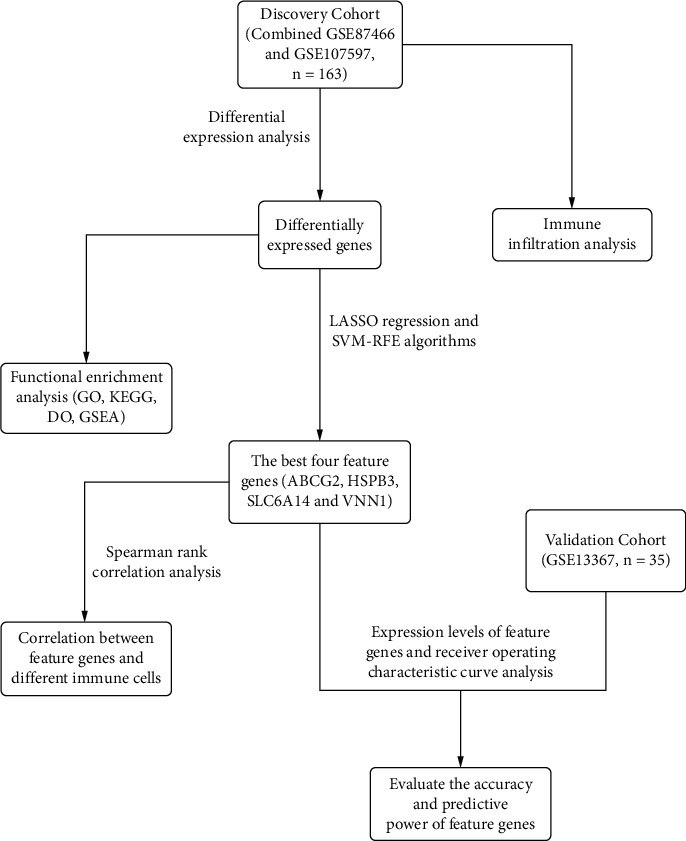
Flowchart of the analysis process.

The flowchart for our study was shown in Figure 1. The GSE87466, GSE107597, and GSE13367 datasets were downloaded from the NCBI Gene Expression Synthesis (GEO) database. The GSE87466 dataset was based on the GPL13158 platform of Affymetrix HT HG-U133+ PM Array Plate, which included 87 UC and 21 normal control tissues. The GSE107597 dataset was based on the GPL15207 platform of Affymetrix Human Gene Expression Array, which included 76 UC and 45 normal control tissues. The “limma R” package and the “sva R” package were used to merge two datasets which served as a discovery cohort. The GSE13367 dataset that included 35 UC and 23 normal control tissues was based on the GPL570 platform of Affymetrix Human Genome U133 Plus 2.0 Array, which served as a validation cohort.

### 2.2. Identification of DEGs and Functional Enrichment Analysis

Using false discovery rate (FDR) < 0.05 and fold change (FC) ≥ 2 or ≤-2 as test criteria, the “limma R” package was used to detect differentially expressed genes. Then, the “clusterProfiler R” package and “DOSE R” package were used for disease ontology (DO) enrichment analysis, GO function enrichment analysis, and KEGG pathway enrichment analysis, which were used to study the potential biological functions of these DEGs. Furthermore, GSEA software (version: 4.1.0) was used for GSEA analysis, and “c2.cp.kegg.v7.0.symbols.gmt” was selected as the reference gene set to explore important functional pathways between the UC and control group.

### 2.3. Potential Biomarker Screening

The least absolute shrinkage and selection operator (LASSO) is a regression analysis algorithm that improves prediction accuracy and selects the best features for high-dimensional data [10]. The “glmnet R” package was used to perform the LASSO regression algorithm to identify genes that were significantly associated with the discrimination of UC and normal tissues. Support vector machine recursive feature elimination (SVM-RFE) is a machine learning method based on support vector machines. It is used to find the best variables by deleting the feature vectors generated by SVM. Compared to the other machine learning methods, SVM is very powerful at recognizing subtle patterns in complex datasets and widely used for classification and regression [11, 12]. The “e1071 R” package was used to perform SVM algorithm to select the most significant feature genes. Finally, we took the intersection of genes from LASSO regression and SVM-RFE algorithms to obtain the best feature genes, which were used to construct regression models and further verified its expression levels in the GSE13367 dataset.

### 2.4. Immune Infiltration Characteristics in UC

CIBERSORT was used to calculate the relative proportions of 22 immune cells in the UC gene expression profile. The “vioplot R” package was used to compare the differences in the levels of 22 immune cells between the UC and the normal control group. The “corrplot R” package was used to calculate the level of 22 kinds of immune cells in each sample and draw a correlation heatmap which revealed the correlation of 22 kinds of immune cells.

### 2.5. Correlation Analysis between Feature Genes and Immune Cells

In R software, the Spearman rank correlation analysis was used to explore the correlation between the selected feature genes and different immune cells, and the analysis results were visualized and drawn by the “ggplot2 R” package.

### 2.6. Statistical Analysis

All statistical analysis was performed by R software (version 4.0.3). The Wilcoxon test was used for comparison between two groups. Kruskal-Wallis tests were used for comparison between two or more groups. Student's *t*-test was used for normally distributed variables and Mann–Whitney *U* test was used for variables with abnormal distribution. *P* < 0.05 was considered statistically significant.

## 3. Results

### 3.1. Identification of DEGs in UC

**Figure 2:**
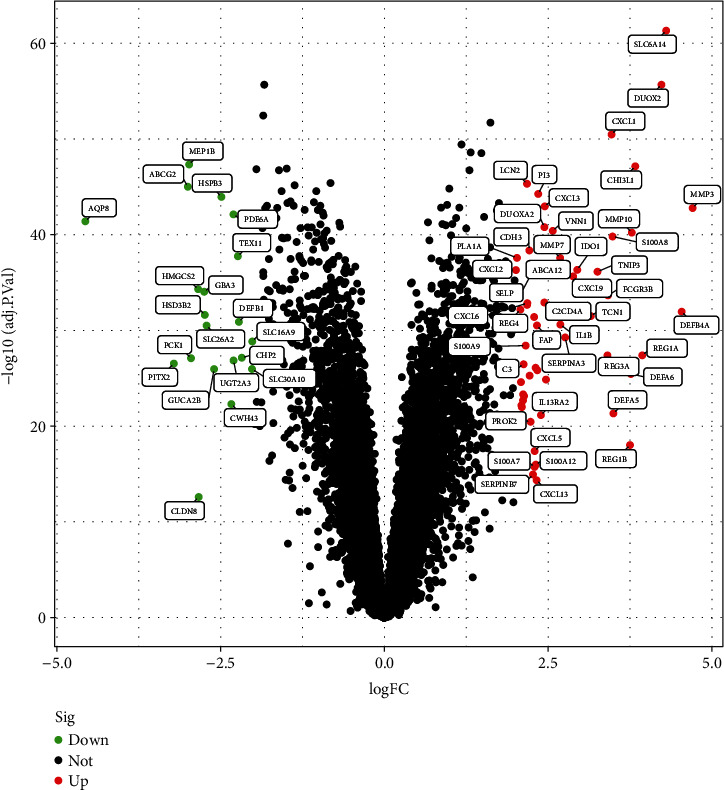
DEGs between UC and normal control tissues (LogFC is log_2_fold change. adj.*P*.Value is the adjusted *P* value, which is more reliable. Since the smaller the *P* value was, the more significant the difference was, then, −log_10_adj.*P*.Value transformation was carried out, and the larger the transformation value was, the more significant the difference was.)

**Figure 3:**
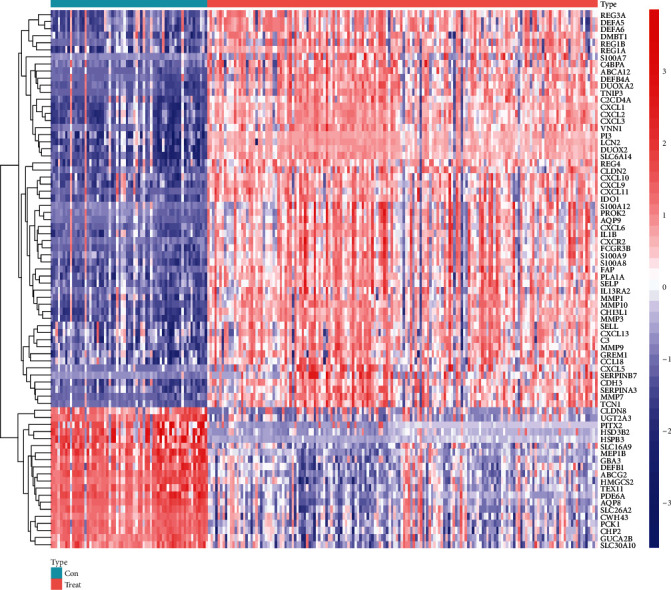
The heatmap of the DEGs.

The data used for analysis came from the two datasets (GSE87466 and GSE107597), including a total of 163 UC and 66 normal control tissues. All expression values were standardized, and a total of 76 DEGs were identified at last (the analysis results were shown in Table 1 of the Supplementary Material), among which 56 genes were upregulated and 20 genes were downregulated (Figure 2). Moreover, the heatmap (Figure 3) has shown the expression of these DEGs in each sample.

### 3.2. Functional Enrichment Analysis

**Figure 4:**
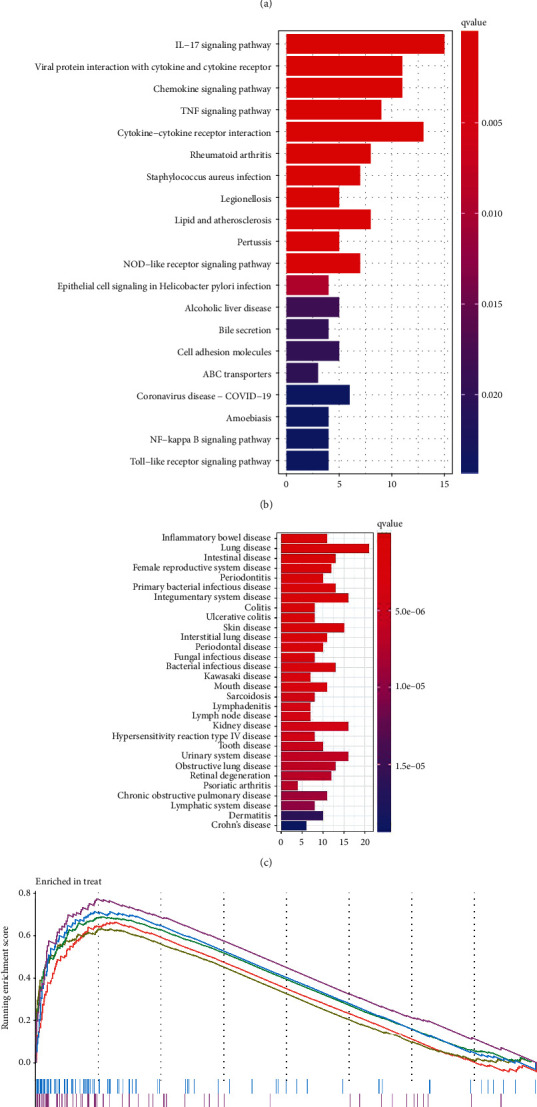
(a) The GO enrichment analysis of DEGs. (b) The KEGG enrichment analysis of DEGs. (c) The DO enrichment analysis of DEGs. (d) The GSEA enrichment analysis of DEGs in the UC group.

GO enrichment analysis results (Figure 4(a)) showed that DEGs were mainly associated with humoral immune response, antimicrobial humoral response, secretory granule lumen, cytokine receptor binding, G protein-coupled receptor binding, receptor-ligand activity, etc. KEGG enrichment analysis results (Figure 4(b)) showed that DEGs were mainly involved in IL-17 signaling pathway, cytokine-cytokine receptor interaction, viral protein interaction with cytokine and cytokine receptor, chemokine signaling pathway, etc. The DO pathway enrichment analysis results (Figure 4(c)) showed that diseases enriched by DEGs mainly included inflammatory bowel disease, lung disease, intestinal disease, and intrinsic system disease. In the GSEA analysis, the pathways enriched in the UC group (Figure 4(d)) mainly included “CELL_ADHESION_MOLECULES_CAMS,” “CHEMOKINE_SIGNALING_PATHWAY,” and “CYTOKINE_CYTOKINE_RECEPTOR_INTERACTION.” In short, functional enrichment analysis indicates that these DEGs are mainly involved in immune response, inflammation, chemokine pathways, and cytokine receptor interactions.

### 3.3. Screening and Verification of Potential Biomarkers

**Figure 5:**
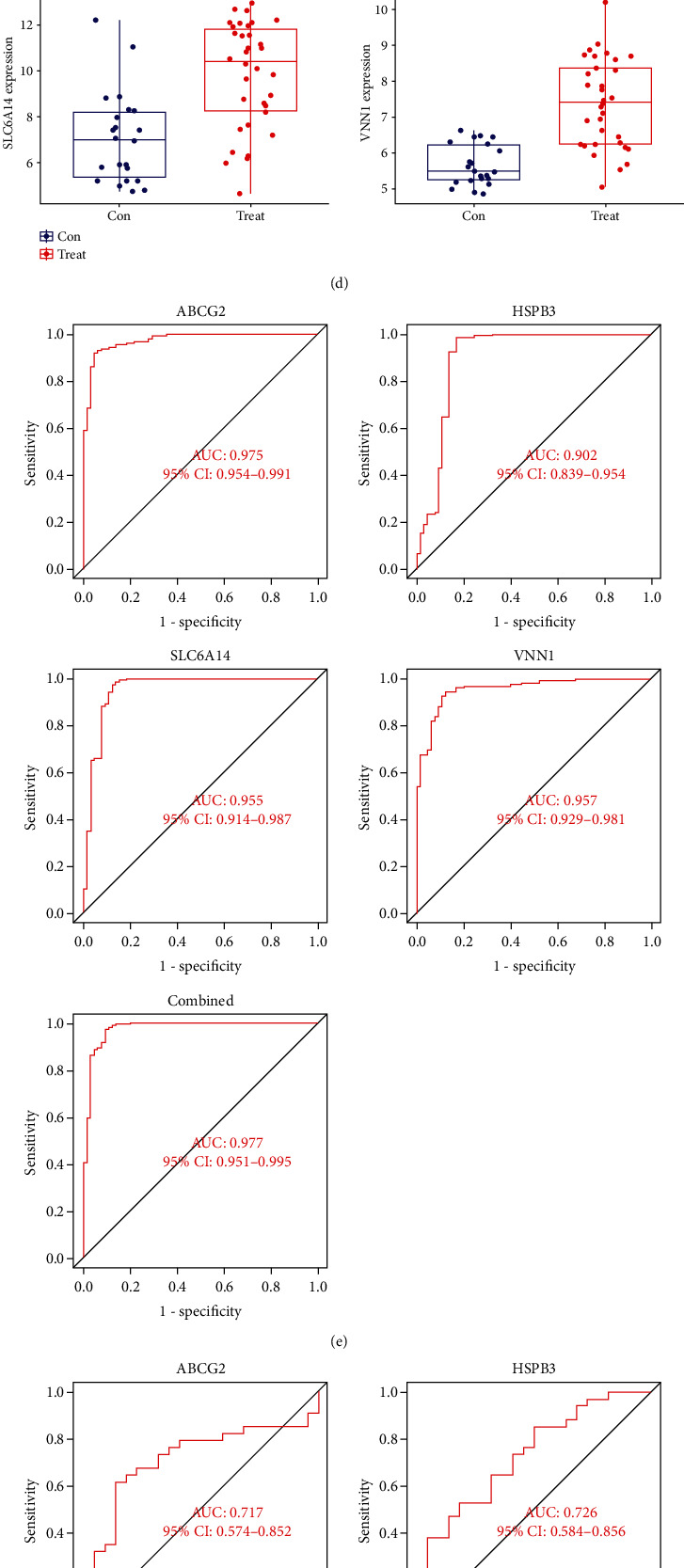
(a) The LASSO regression model used 10-fold cross-validation and the minimum absolute shrinkage criterion to identify the optimal penalty coefficient *λ*. (b) Screening out feature genes by SVM-RFE algorithm. (c) Venn diagram of intersection feature genes between the LASSO regression model and SVM-RFE algorithm. (d) The expression levels of the four genes between UC group (red) and normal control group (blue) in the validation cohort. (e) ROC curve of the four feature genes in the discovery cohort. (f) ROC curve of the four feature genes in the validation cohort.

Two different algorithms were used to screen out the best biomarkers for diagnosing UC. 26 genes were identified by the LASSO regression algorithm (Figure 5(a)), and 11 genes were identified by the SVM-RFE algorithm (Figure 5(b)). Eventually, we got four overlapping features genes between these two algorithms (Figure 5(c)): ABCG2, HSPB3, SLC6A14, and VNN1, among which the expression of ABCG2 and HSPB3 was downregulated, and the expression of SLC6A14 and VNN1 was upregulated. The predictive model was constructed by using the logistic regression algorithm. In order to further evaluate the accuracy and predictive power of the four genes as diagnostic biomarkers, the GSE13367 dataset was used for verification. The results showed that in UC, the expression levels of ABCG2 and HSPB3 were significantly lower than normal control group, while the expression levels of SLC6A14 and VNN1 were significantly higher than normal control group (Figure 5(d)). The area under the ROC curve (AUC) was used to evaluate the predictive value of four genes for diagnosis of UC in the two cohorts. In the discovery cohort (Figure 5(e)), the AUC of ABCG2 was 0.975 (95% CI: 0.954~0.991), the AUC of HSPB3 was 0.902 (95% CI: 0.839 to 0.954), the AUC of SLC6A14 was 0.955 (95% CI: 0.914 to 0.987), and the AUC of VNN1 was 0.957 (95% CI: 0.929~0.987). Then, when these four genes were fitted into one variable, and the AUC was 0.977 (95% CI: 0.951~0.995). The accuracy and *F*1-score of the predictive model constructed by four genes were 95.20% and 95.20%. In the validation cohort (Figure 5(f)), the AUC of ABCG2 was 0.717 (95% CI: 0.574 to 0.852), the AUC of HSPB3 was 0.726 (95% CI: 0.584 to 0.856), the AUC of SLC6A14 was 0.811 (95% CI: 0.691) ~0.922), and the AUC of VNN1 is 0.886 (95% CI: 0.797~0.961). Moreover, when the four genes were fitted into one variable, the AUC was 0.889 (95% CI: 0.797~0.961), and the accuracy of this model was 87.4%, and similarly, the *F*1-score was 87.4%. The above analysis results showed that these four feature genes had great diagnostic value and predictive power for distinguishing UC from the normal control group.

### 3.4. Immune Infiltration Analysis

**Figure 6:**
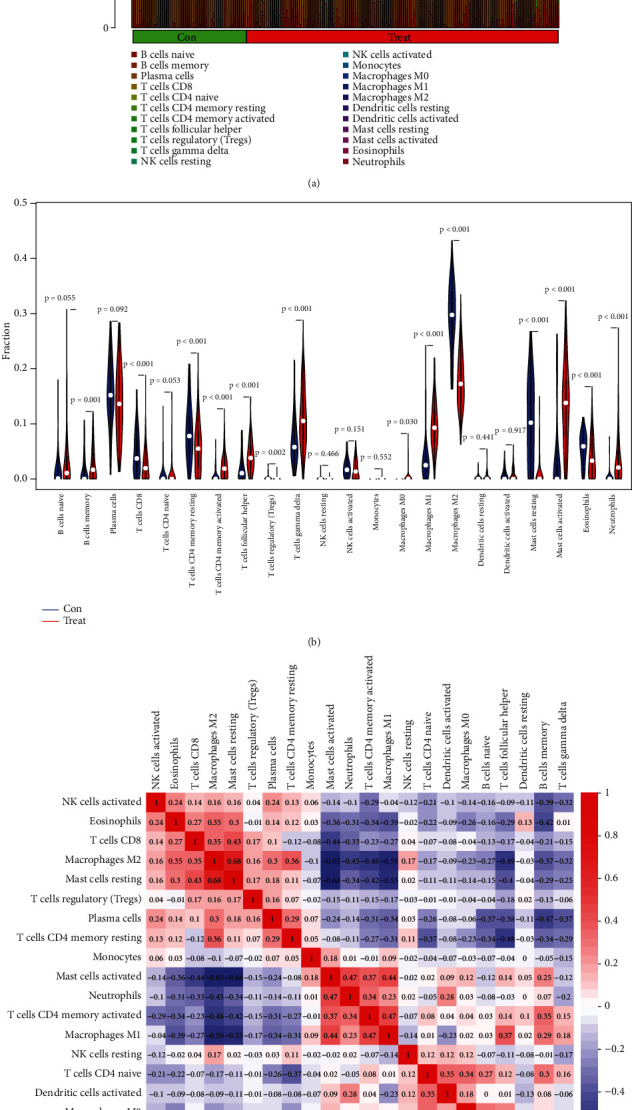
(a) The relative percentage of 22 immune cells in each sample of the discovery cohort. (b) The difference in immune infiltration between the UC and normal control groups with red representing the UC group and blue representing the normal control group. (c) The correlation heatmap between 22 immune cells with red representing positive correlation and blue representing negative correlation. The darker the color, the stronger the correlation.

CIBERSORT was used to analyze the immune infiltration between the UC and the normal control group. Firstly, the percentage of 22 kinds of immune cells in each sample was calculated (Figure 6(a)). Secondly, according to the vioplot (Violin Plot) of the difference in immune cell infiltration between the UC and normal control groups (Figure 6(b)), there was a high proportion of memory B cells, *γδ* T cells, activated mast cells, M1 macrophages, and neutrophils in UC; however, in normal control group, there was a high proportion of M2 type macrophages, resting mast cells, eosinophils, and CD8+ T cells. The correlation heatmap of immune cells (Figure 6(c)) showed that resting mast cells were positively correlated with M2 type macrophages (*r* = 0.68) and CD8+ T cells (*r* = 0.43). Activated mast cells were positively correlated with neutrophils (*r* = 0.47), activated CD4+ memory T cells (*r* = 0.37), and M1 macrophages (*r* = 0.44). Follicular helper T cells were positively correlated with M1 type macrophages (*r* = 0.37) and naive B cells (*r* = 0.51); however, memory B cells were negatively correlated with eosinophils (*r* = −0.42), activated NK cells (*r* = −0.39), and plasma cells (*r* = −0.47). Based on the above analysis results, there were significant differences in immune cell infiltration between the UC and normal control groups.

### 3.5. Correlation between Four Feature Genes and Immune Cell Infiltration

**Figure 7:**
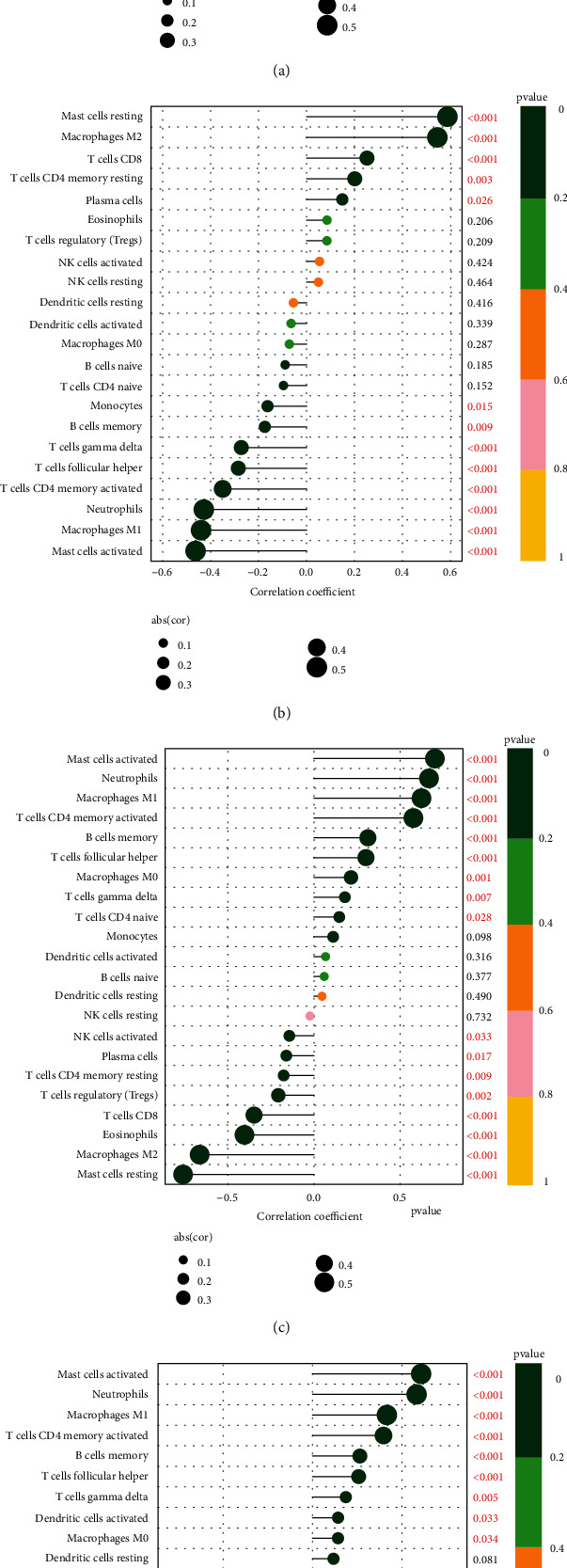
(a) The correlation between ABCG2 and immune cells. (b) The correlation between HSPB3 and immune cells. (c) The correlation between SLC6A14 and immune cells. (d) The correlation between VNN1 and immune cells.

Correlation analysis showed that ABCG2 (Figure 7(a)) was positively correlated with M2 type macrophages (*r* = 0.78, *P* < 0.001), resting mast cells (*r* = 0.69, *P* < 0.001), eosinophils (*r* = 0.49, *P* < 0.001), and plasma cells (*r* = 0.25, *P* < 0.001) but was negatively correlated with M1 type macrophages (*r* = −0.64, *P* < 0.001), neutrophils (*r* = −0.63, *P* < 0.001), activated mast cells (*r* = −0.60, P <0.001), activated CD4+ memory T cells (*r* = −0.53, *P* < 0.001), and follicular helper T cells (*r* = −0.48, *P* < 0.001). HSPB3 (Figure 7(b)) was positively correlated with M2 type macrophages (*r* = 0.55, *P* < 0.001), resting mast cells (*r* = 0.59, *P* < 0.001), and CD8+ T cells (*r* = 0.25, *P* < 0.001) but was negatively correlated with M1 type macrophages (*r* = −0.44, *P* < 0.001), neutrophils (*r* = −0.43, *P* < 0.001), activated mast cells (*r* = −0.46, *P* < 0.001), activated CD4+ memory T cells (*r* = −0.35, *P* < 0.001), and gamma delta T cells (*r* = −0.27, *P* < 0.001). SLC6A14 (Figure 7(c)) was positively correlated with activated mast cells (*r* = 0.70, *P* < 0.001), neutrophils (*r* = 0.67, *P* < 0.001), M1 macrophages (*r* = 0.62, *P* < 0.001), and activated CD4+ memory T cells (*r* = 0.58, *P* < 0.001) but was negatively correlated with M2 type macrophages (*r* = −0.66, *P* < 0.001), resting mast cells (*r* = −0.76, *P* < 0.001), eosinophils (*r* = −0.40, *P* < 0.001), and CD8+ T cells (*r* = −0.35, *P* < 0.001). VNN1 (Figure 7(d)) was positively correlated with activated mast cells (*r* = 0.61, *P* < 0.001), neutrophils (*r* = 0.58, *P* < 0.001), M1 macrophages (*r* = 0.42, *P* < 0.001), activated CD4+ memory T cells (*r* = 0.40, *P* < 0.001), and memory B cells (*r* = 0.26, *P* < 0.001) but was negatively correlated with resting mast cells (*r* = −0.72, *P* < 0.001), M2 type macrophages (*r* = −0.53, *P* < 0.001), CD8+ T cells (*r* = −0.35, *P* < 0.001), and regulatory T cells (*r* = −0.48, *P* = 0.002). In general, the four feature genes (ABCG2, HSPB3, SLC6A14, and VNN1) were all related to immune cell infiltration.

## 4. Discussion

Although the early diagnosis and treatment of UC have been greatly improved in the past ten years, because of unclear specific pathogenesis, the main purpose of treatment is to induce and maintain remission, and the long-term prognosis is not optimistic [4–6]. By looking for new potential biomarkers at the gene level and analyzing the characteristics of UC immune cell infiltration, it will have a very beneficial impact on the early diagnosis and prognosis evaluation of UC patients and can also provide insights for finding new treatment targets [7, 13, 14]. In this study, we had identified a total of 76 DEGs based on the gene expression dataset of the UC and normal control groups, among which 56 genes were upregulated and 20 genes were downregulated. Multiple functional enrichment analysis showed that these DEGs were significantly related to immune response, chemokine signaling pathway, IL-17 signaling pathway, and cytokine receptor interaction. Then we selected four best feature genes (ABCG2, HSPB3, SLC6A14 and VNN1) based on two machine learning algorithms (LASSO regression model and SVM-RFE algorithm). Meanwhile, the high expression of ABCG2 and HSPB3 may play a protective role in the prognosis of UC, while the high expression of SLC6A14 and VNN1 may promote the occurrence and development of UC.

The overexpression of ATP-binding cassette transporter G2 (ABCG2), also known as breast cancer resistance protein (BCRP), can make cancer cells resistant to chemotherapeutic drugs such as mitoxantrone and doxorubicin with ABCG2 as the substrate [15]. Meanwhile, ABCG2 is identified as a physiological important uric acid transporter, and its dysfunction can increase the risk of gout and hyperuricemia [16]. ABCG2 is also expressed in a variety of normal tissues (e.g., intestinal epithelium, brush border membrane of proximal tubules of kidney, and bile duct membrane of hepatocytes in the liver) [17–19], and recent studies [19, 20] suggested that the expression of ABCG2 in active UC is significantly reduced, and it is negatively correlated with the expression of microRNA (involved in posttranscriptional gene regulation and playing a key regulatory function in the pathogenesis of inflammatory bowel disease), which may be because the proinflammatory cytokines are involved in the regulation of transporters. Small heat shock protein (sHSP) has a variety of functions, including cell signaling, cell differentiation and apoptosis, activating immune cells, and stimulating anti-inflammatory and antiplatelet reaction [21–23]. Heat shock protein 3(HSPB3) is the third member of human sHSP family, which is mainly expressed in skeletal muscles and smooth muscles [22, 23]. At present, the function of HSPB3 in UC has not yet been reported, but Kalioraki et al. [23] pointed out that the expression of HSPB3 is downregulated in most colorectal malignancies. UC has the potential risk of canceration, and HSPB3 may play an important role in this process [24]. The expression of solute carrier family 6 member 14 (SLC6A14), also known as amino acid transporter B^0,+^, is upregulated in active UC and colorectal cancer [25–27]. The mechanism of SLC6A14 in UC is currently unclear, but as a high-capacity and condensed amino acid transporter, it can ensure the amino acid nutrition of cells, activate mTOR signal pathway, prevent oxidative stress, and make the cells proliferate rapidly, so it plays an important role in the canceration of UC [27, 28]. In addition, SLC6A14 is involved in the host's antibacterial response and affects the gut microbiota [29], and some studies [30, 31] have pointed out that the use of intestinal microorganism-based immunotherapy to carry out immunity stimulation can improve the intestinal barrier function. Vanin-1 (VNN1) is a glycosyl-phosphatidyl-inositol anchored pantothenase, which can catalyze the hydrolysis of pantetheine to cysteamine and pantothenic acid, thereby playing a significant role in oxidative stress, inflammation and cell migration [32–34]. Most studies [25, 35] proposed that the expression of VNN1 is upregulated in UC; in addition, stimulating the expression of VNN1 will promote the occurrence of colorectal carcinoma. Therefore, the four feature genes screened in this study are related to cell signaling, inflammation, and immune response and may be involved in the occurrence and progression of UC.

Through CIBERSORT algorithm analysis, it was found that UC was significantly different from the immune cellular infiltration in the control group, and several immune cell subtypes are closely related to the biological process of UC. Recent studies also confirmed that immune cell infiltration exerts an important role in the genesis and development of UC: activated neutrophils will accumulate in the blood and colon tissues of patients with active UC, and the expression of costimulatory molecules will be enhanced in dendritic cells of these patients [4, 36, 37]. The inflammatory environment of UC potentially improves the survival of neutrophilic granulocytes through HIF-1 and hypoxia. This increased survival rate will intensify inflammation and tissue injury in a variety of ways, including the release of serine, matrix metalloproteinases, reactive oxygen, and proinflammatory cytokines [37]. The IgG1 antibodies in the blood of patients with UC increase disproportionately; hence, B cells also play a certain role in the pathogenesis of UC [37, 38]. Besides, the imbalance of follicular helper T cells and follicular regulatory T cells is related to the disease activities of UC [39]. In our research, through correlation analysis, it can be seen that the four feature genes screened out are all related to the immune cell infiltration of UC, which is expected to become the direction of future research.

In recent years, the combination of microarray technology, bioinformatics analysis, and different machine learning algorithms for biomarker screening, diagnostic prediction, and prognosis assessment of complex diseases has become a hot topic, and the method of computational biology can also provide scope and basis for further basic experimental design [9, 40]. In this study, the overlap of the LASSO model and SVM-RFE algorithm was used to screen potential biomarkers of UC, which was little reported in the past. However, due to the limited data in this study, more external data should be used for verification, and it is also necessary to evaluate the reliability of the results through clinical sample experiments. What is more, prospective clinical studies will be designed in the future to evaluate the practical use of these potential biomarkers.

## 5. Conclusion

In summary, through three GEO datasets and two machine learning algorithms, ABCG2, HSPB3, SLC6A14, and VNN1 were identified as potential biomarkers of UC. The four biomarkers are involved with multiple biological processes such as cell signal transduction and inflammation; at the same time, they are related to immune cell infiltration, which may become new treatment targets for UC in the future.

## Data Availability

All raw data of our research are downloaded from the NCBI Gene Expression Synthesis (GEO) database(https://www.ncbi.nlm.nih.gov/geo/), which are publicly available databases. Accession numbers: GSE87466, GSE107597, and GSE13367.

## Supplementary Materials

Supplementary Materials 76 differentially expressed genes.
