# Supplementary material for: Identification of Potential Biomarkers and Immune Infiltration Characteristics in Ulcerative Colitis by Combining Results from Two Machine Learning Algorithms
Source: Comput Math Methods Med. 2022 Aug 1;2022:5412627. doi: 10.1155/2022/5412627 (PMC9359832; doi:10.1155/2022/5412627)
Supplement: Supplementary Materials — 76 differentially expressed genes. [file 5412627.f1.pdf]

Table 1. 76 differentially expressed genes

Id	LogFC	AveExpr	t	P.Value	adj.P.Val	B
SLC6A14	4.302268524	9.115340198	24.64955163	3.03E-66	4.84E-62	140.2245259
DUOX2	4.230123346	10.17895667	22.59537532	4.03E-60	2.15E-56	126.2603611
CXCL1	3.467955422	10.19726234	20.81044758	1.27E-54	3.39E-51	113.7085205
MEP1B	-2.980991686	6.580204934	-19.74506526	2.88E-51	4.61E-48	106.0458233
CHI3L1	3.830226769	8.571360031	19.67117139	4.95E-51	7.19E-48	105.5099646
LCN2	2.177201415	12.31283125	19.04056121	5.08E-49	4.77E-46	100.9152778
ABCG2	-3.001555655	8.434906357	-18.92822728	1.16E-48	9.77E-46	100.0928737
PI3	2.348315338	12.50319284	18.66784289	7.97E-48	5.54E-45	98.1822339
HSPB3	-2.488590389	5.124032542	-18.56629201	1.69E-47	1.13E-44	97.4354723
CXCL3	2.448532879	9.10075496	18.21593582	2.28E-46	1.11E-43	94.85247642
MMP3	4.706115253	9.232607057	18.14737618	3.80E-46	1.64E-43	94.3458593
PDE6A	-2.305178546	6.180383806	-17.92267295	2.03E-45	7.54E-43	92.68288075
AQP8	-4.566580987	8.804840617	-17.68093169	1.24E-44	4.03E-42	90.88962526
DUOXA2	2.442938634	7.44174433	17.47124124	5.95E-44	1.64E-41	89.33081128
VNN1	2.570455265	7.907832819	17.33739538	1.62E-43	3.99E-41	88.33429434
MMP10	3.777462456	7.693087841	17.27863042	2.52E-43	6.10E-41	87.89641441
S100A8	3.483325528	10.14058465	17.1441801	6.92E-43	1.56E-40	86.89377857
CDH3	2.212754855	7.566581714	16.67090613	2.44E-41	4.43E-39	83.35631725
TEX11	-2.237931154	7.883188383	-16.48202183	1.02E-40	1.78E-38	81.94136037
PLA1A	2.023973574	7.525765731	16.42625897	1.55E-40	2.63E-38	81.52332889

MMP7	2.683214577	7.307734053	16.41861095	1.64E-40	2.73E-38	81.46598439
ABCA12	2.222039506	6.182074595	16.08401168	2.06E-39	2.63E-37	78.95493466
IDO1	2.944851685	9.126208216	15.99915374	3.91E-39	4.73E-37	78.31747524
CXCL2	2.008495086	8.10860007	15.99102329	4.15E-39	4.95E-37	78.25638673
TNIP3	3.253313511	7.296218661	15.93450942	6.37E-39	7.37E-37	77.83171197
SELP	2.253342983	8.356554198	15.8986018	8.36E-39	9.34E-37	77.56183474
CXCL9	2.882114723	9.468519952	15.77207555	2.18E-38	2.27E-36	76.61059976
HMGCS2	-2.844306868	9.856478128	-15.33546741	5.96E-37	4.91E-35	73.32551598
GBA3	-2.751837946	7.390546903	-15.24333017	1.20E-36	9.16E-35	72.63190678
FCGR3B	3.413876685	7.439462718	15.12022929	3.05E-36	2.21E-34	71.70509871
C2CD4A	2.441441225	7.474495863	14.88409593	1.83E-35	1.22E-33	69.9271614
CXCL6	2.18235578	5.888215727	14.860617	2.18E-35	1.43E-33	69.75038379
MMP9	2.178516304	8.74617363	14.79941367	3.47E-35	2.15E-33	69.28958469
REG4	2.076138341	10.07361873	14.63449651	1.21E-34	6.79E-33	68.04807455
DEFB4A	4.538669665	8.565108952	14.56449492	2.06E-34	1.11E-32	67.52119158
HSD3B2	-2.742752806	5.433688894	-14.45692525	4.66E-34	2.41E-32	66.71169389
TCN1	3.151884305	6.733395167	14.4158377	6.36E-34	3.19E-32	66.40255261
CLDN2	2.286323405	7.123965441	14.38467264	8.05E-34	3.97E-32	66.1680911
DEFB1	-2.222298202	8.00550437	-14.22083842	2.79E-33	1.28E-31	64.93590914
IL1B	2.686475142	8.9498793	14.12916976	5.58E-33	2.45E-31	64.24679877
FAP	2.329372282	6.970362885	14.09773086	7.08E-33	3.04E-31	64.01052021
SLC26A2	-2.715708582	9.738378639	-14.09448498	7.25E-33	3.11E-31	63.98612773

SERPINA3	2.760106224	8.010844145	13.69740971	1.46E-31	5.43E-30	61.00522384
SLC16A9	-2.017357664	7.08052874	-13.56280169	4.04E-31	1.43E-29	59.99635768
S100A9	2.156692617	8.163179247	13.42310465	1.16E-30	3.85E-29	58.95042317
REG1A	3.936939638	11.10100486	13.09070265	1.41E-29	4.03E-28	56.46678977
REG3A	3.405627322	7.882571969	13.08798161	1.44E-29	4.10E-28	56.44649179
CHP2	-2.177653634	10.31628004	-13.00947525	2.60E-29	7.12E-28	55.86111673
PCK1	-2.952769816	8.167171436	-12.99220601	2.96E-29	8.05E-28	55.73241709
UGT2A3	-2.3035197	6.197190775	-12.91348887	5.35E-29	1.38E-27	55.14609047
PITX2	-3.215161383	5.339451449	-12.80727845	1.19E-28	2.90E-27	54.35583328
C3	2.125896569	11.10662304	12.78226055	1.43E-28	3.45E-27	54.16983643
MMP1	2.308524588	10.30610775	12.66280642	3.50E-28	7.99E-27	53.28256537
SLC30A10	-2.021834152	6.900569943	-12.62075415	4.79E-28	1.08E-26	52.97054554
GUCA2B	-2.600398407	8.801762128	-12.61879603	4.87E-28	1.09E-26	52.95602095
CXCL10	2.334847426	9.014796811	12.57845474	6.58E-28	1.45E-26	52.65687284
DEFA6	3.76406586	9.500766167	12.45216724	1.69E-27	3.55E-26	51.72150237
CXCL11	2.217388826	6.079016269	12.39332883	2.62E-27	5.36E-26	51.28629973
AQP9	2.467019225	6.336535982	12.26004525	7.06E-27	1.37E-25	50.30192821
DMBT1	2.085032177	9.896106793	12.17383644	1.34E-26	2.53E-25	49.66636406
SELL	2.117919225	8.382746414	11.75366992	3.00E-25	4.77E-24	46.58277374
CXCR2	2.13255243	7.155008141	11.69592995	4.60E-25	7.16E-24	46.16099254
CCL18	2.116507073	9.501758423	11.54373074	1.41E-24	2.08E-23	45.05168343
CWH43	-2.337642824	8.54641622	-11.41861025	3.52E-24	4.98E-23	44.14255221

GREM1	2.091434778	8.543844789	11.37100161	4.99E-24	6.93E-23	43.7973201
C4BPA	2.098080899	8.595033159	11.31295642	7.62E-24	1.04E-22	43.37694059
DEFA5	3.496906882	9.80893478	11.09049409	3.85E-23	4.75E-22	41.7714457
IL13RA2	2.390028317	6.950149529	11.03353208	5.82E-23	7.05E-22	41.36185141
PROK2	2.23214798	6.005001233	10.80063591	3.13E-22	3.51E-21	39.69390356
REG1B	3.750316896	8.320661009	9.972201521	1.14E-19	9.49E-19	33.85983559
CXCL5	2.293851227	5.621235211	9.75594817	5.14E-19	4.02E-18	32.36582938
S100A12	2.311150026	6.207958379	9.25842517	1.58E-17	1.05E-16	28.9810505
S100A7	2.292920393	6.783856731	9.173312511	2.81E-17	1.83E-16	28.40985159
SERPINB7	2.268365138	5.172648824	8.88987538	1.89E-16	1.13E-15	26.52544115
CXCL13	2.323418322	8.827605634	8.673190165	7.99E-16	4.51E-15	25.10414314
CLDN8	-2.835571665	7.302042692	-8.024317158	5.36E-14	2.55E-13	20.95743256

logFC = log2fold change

AveExpr = average expression

t = the adjusted T-test results

B = log value of standard deviation after Bayes adjustment
